# Abnormal Chondrocyte Apoptosis in the Cartilage Growth Plate is Influenced by Genetic Background and Deletion of CHOP in a Targeted Mouse Model of Pseudoachondroplasia

**DOI:** 10.1371/journal.pone.0085145

**Published:** 2014-02-18

**Authors:** Katarzyna A. Piróg, Andreja Irman, Siobhan Young, Poonam Halai, Peter A. Bell, Raymond P. Boot-Handford, Michael D. Briggs

**Affiliations:** 1 Institute of Genetic Medicine, Newcastle University, Newcastle Upon Tyne, United Kingdom; 2 Wellcome Trust Centre for Cell-Matrix Research, University of Manchester, Manchester, United Kingdom; University of Maryland School of Medicine, United States of America

## Abstract

Pseudoachondroplasia (PSACH) is an autosomal dominant skeletal dysplasia caused by mutations in cartilage oligomeric matrix protein (COMP) and characterised by short limbed dwarfism and early onset osteoarthritis. Mouse models of PSACH show variable retention of mutant COMP in the ER of chondrocytes, however, in each case a different stress pathway is activated and the underlying disease mechanisms remain largely unknown. T585M COMP mutant mice are a model of moderate PSACH and demonstrate a mild ER stress response. Although mutant COMP is not retained in significant quantities within the ER of chondrocytes, both BiP and the pro-apoptotic ER stress-related transcription factor CHOP are mildly elevated, whilst bcl-2 levels are decreased, resulting in increased and spatially dysregulated chondrocyte apoptosis. To determine whether the abnormal chondrocyte apoptosis observed in the growth plate of mutant mice is CHOP-mediated, we bred T585M COMP mutant mice with CHOP-null mice to homozygosity, and analysed the resulting phenotype. Although abnormal apoptosis was alleviated in the resting zone following CHOP deletion, the mutant growth plates were generally more disorganised. Furthermore, the bone lengths of COMP mutant CHOP null mice were significantly shorter at 9 weeks of age when compared to the COMP mutant mice, including a significant difference in the skull length. Overall, these data demonstrate that CHOP-mediated apoptosis is an early event in the pathobiology of PSACH and suggest that the lack of CHOP, in conjunction with a COMP mutation, may lead to aggravation of the skeletal phenotype via a potentially synergistic effect on endochondral ossification.

## Introduction

Pseudoachondroplasia (PSACH) is an autosomal dominant skeletal dysplasia resulting from mutations in cartilage oligomeric matrix protein (COMP), a large pentameric glycoprotein present in cartilage, bone, skeletal muscle, tendon and ligament [1], [2]. PSACH manifests with short-limbed dwarfism, joint laxity and early onset osteoarthritis [3], [4]. COMP is thought to act as a bridging molecule in the cartilage extracellular matrix (ECM) and interacts with a number of other structural ECM molecules such as matrilin-3 [5], type IX collagen [6], type II collagen [7] and aggrecan [8] as well as signalling receptors such as integrins [9], [10]. PSACH-causing mutations in COMP cluster in two distinct regions of the molecule, the thrombospondin type 3 (T3-COMP) repeats and the C-terminal (CTD-COMP) globular domain [11].

Type 3 repeat mutations account for the majority PSACH cases and are all believed to result in misfolding of the mutant protein and its retention in the endoplasmic reticulum (ER) [12], [13]. Analysis of the growth plate in a targeted mouse model of T3-COMP PSACH with the common p.D469del mutation demonstrated increased and dysregulated chondrocyte apoptosis and decreased proliferation. However, there was no transcriptional evidence of a conventional ER stress response in mutant chondrocytes [14] and an aggregated protein response (APR) was proposed as an alternative mechanism.

In contrast, the CTD-COMP mutations, which account for a smaller percentage of PSACH often allow the secretion of the mutant protein [15], [16]. In a T585M CTD-COMP targeted mouse model the mild stress response leads to decreased chondrocyte proliferation and a dysregulated increase in apoptosis [16]. The mild ER stress is characterised by a transcriptional increase in several ER markers including BiP and the pro-apoptotic transcription factor CHOP. We therefore hypothesised that the abnormal chondrocyte apoptosis in the T585M COMP mouse growth plate was CHOP-mediated as a direct result of the ER stress caused by the folding and trafficking of mutant T585M COMP protein [16].

CHOP [also known as DDIT3 (DNA Damage Inducible Transcript 3) and GADD153 (Growth Arrest and DNA-Damage inducible gene)] is an ER stress inducible leucine zipper transcription factor associated with ER stress related apoptosis [17]–[19]. CHOP can be activated via the PERK and ATF6 unfolded protein response (UPR) pathways and acts to decrease the levels of the anti-apoptotic protein bcl-2, which subsequently renders the cells more susceptible to programmed cell death [20], [21]. Mouse embryonic fibroblasts derived from mice that are null for CHOP are resistant to ER stress induced apoptosis [22] and CHOP deficiency promotes cell survival in an ER stress related model of type 2 diabetes [23]. CHOP can also be upregulated in oxidative stress conditions independently of ER stress [24]. CHOP can form heterodimers with other molecules such as C/EBPβ and LAP and act as a transdominant negative inhibitor of C/EBPβ signalling [17], it may also modulate transcriptional activity of other genes via its interactions with fos/jun and atf proteins [25], [26].

To determine whether the increase in chondrocyte apoptosis in the CTD-COMP mouse model of mild PSACH was CHOP mediated we crossed this mouse model with CHOP null mice and analysed the resulting phenotype. In this paper we confirm that CHOP plays an important role in bone biology, but more importantly we demonstrate that the dysregulation of apoptosis in the proliferative zone of growth plates in T585M COMP mutant mice is not directly mediated by CHOP.

## Materials and Methods

### Generation of mouse lines

CHOP null mice (B6.129S-Ddit3tm1Dron/J) were obtained from the Jackson Laboratory and crossed with homozygous T585M COMP knock-in mice [16] to obtain mice heterozygous for both mutations. These mice were then recrossed with the COMP mutant mice to obtain mice that were homozygous for the T585M COMP mutation and either wild type or knock-out for CHOP. C57BL6/J mice were obtained from Jackson Laboratory. All experiments were approved by the University of Manchester Animal Ethical Review Group and performed in compliance with the Scientific Procedures Act of 1986 and the relevant Home Office (under PPL 40/2884) and Institutional regulations governing animal breeding and handling.

### Bone measurements

Mice of the relevant genotypes (10 per age per genotype) were sacrificed at 3, 6 and 9 weeks of age and X-rayed using a Faxitron MX-20 X-ray machine. A direct phenotypic comparison between mice with different genetic backgrounds may not be biologically relevant due to differences in the basal levels of the quantitative disease parameters [27]. Therefore, genetically matched controls were used for each of the mouse models in this study and in all cases data generated from mutant mice were compared to the relevant controls. Bone lengths were determined using Growbase software (Certus Technology Ltd) and one-way ANOVA and t-test were used for statistical analysis. Hip angles were measured as described previously [16] (Figure S1).

### Histology and immunohistochemistry

Mice were sacrificed at 3 weeks of age and hindlimbs were dissected from surrounding tissues. These were fixed in either 10% neutral buffered formaldehyde (PFA; histology) or 95% ethanol 5% acetic acid (immunohistochemistry) for 48 h in 4°C. Limbs were then decalcified in 20% EDTA pH 7.4 over 2 weeks, wax embedded and cut into 6 µm sections. Haematoxylin/eosin (H&E) staining was used to visualise the general morphology of the tissue using the automated Thermo Shandon stainer.

Immunohistochemistry and BrdU labelling (a real-time measurement of cell proliferation) were performed as described previously [16]. BrdU labelled cells were counted and presented as a percentage of all (DAPI stained) cells in the proliferative zone with One-Way ANOVA used for statistical analysis of these data (Figure S2).

### TUNEL assay

TUNEL assay was performed on PFA fixed sections of 3 week old limbs using the Promega Dead-End Fluorimetric Kit as described previously [16]. The samples were unmasked using citric buffer boil [16] instead of proteinase K unmasking, which can generate false positives [28]. Positive cells labelled with FITC were counted and presented as a percentage of all (DAPI stained) cells in selected zones of the growth plate. One-Way ANOVA was used for statistical analysis of the data.

### Western blotting and mass spectrometry analysis using xiphoid cartilage

The xiphoid process is a well-established model in the proteomic and transcriptomic analysis of chondrodysplasias. It contains a rudimentary growth plate (Figure S3 and S4), which we have previously shown accurately recapitulates the disease pathology seen in mutant tibia, and it can be cleanly and easily dissected [29]. A representative image of the growth plate found in the xiphoid process has been included in Figure S3. Xiphoid processes were extracted from mice of the different genotypes at 3 weeks of age, snap frozen in liquid nitrogen and dismembranated in 100 µl dH_2_O, either separately, or in pooled samples (3 mice per sample). Total protein content in the samples was measured using the BCA assay (Thermo Scientific) and the samples were run for 5 minutes at 200 V into 10% BisTris precast gel (Novex). Gels were stained for 15 min with InstantBlue Coomassie-based stain (Expedeon) and rinsed with distilled water. Gel slices were then excised and washed in acetonitrile for 5 min, dried in a vacuum centrifuge for 15 min and reduced for 1 h (56°C) in 10 mM DTT, 25 mM NH_4_HCO_3_. Samples were cooled to room temperature and DTT was removed by centrifugation.The proteins within the gel were alkylated with 55 mM iodoacetamide in 25 mM NH_4_HCO_3_ for 45 min. The samples were then centrifuged and washed with 25 mM NH_4_HCO_3_ for 10 min, then washed in acetonitrile and dried in a vacuum centrifuge for 15 min. Proteins were digested using 12.5 ng/µl trypsin solution (Sigma) in 25 mM NH_4_HCO_3_ overnight at 37°C. Digested peptides were extracted with 20 mM NH_4_HCO_3_ for 20 min, centrifuged, then extracted twice with 5% formic acid in 50% acetonitrile. The extractions were pooled and concentrated in a vacuum centrifuge before a MALDI-TOF analysis. Data were interrogated using Mascot version 2.2 (Matrix Science, UK) against the UniProt database (version 2011-05) with taxonomy of *Mus musculus* and the following search parameters selected: fragment tolerance: 0.6 Da; parent tolerance: 0.5 Da; fixed modifications allowed: +57 on C (carbamidomethyl), +16 on M (oxidation); max missed cleavages: 1. Mascot search results were validated using Scaffold version 3.3.1 (Proteome Software, Portland, USA) to assign confidence values to peptide/protein matches, where Peptide/Protein Prophet algorithm confidence values of 0.7 and 0.99 were used respectively. Identified proteins were defined as having a number of matched peptide spectra ≥2, and the unweighted spectral count was used as a measure of quantification. These parameters constrained the protein false discovery rate (FDR) to ≤0.2% in all analyses. Four biological replicates were used. The number of spectra identified for each protein were compared between COMP m/m CHOP +/+ and COMP m/m CHOP −/− genotypes using the beta-binomial test in R (version 2.14.2, BetaBinomial package) [30]. A P-value<0.05 was considered significant.

## Results

### Long bone growth in CHOP null mice is normal but their heads are shorter by 9 weeks of age

**Figure 1:**
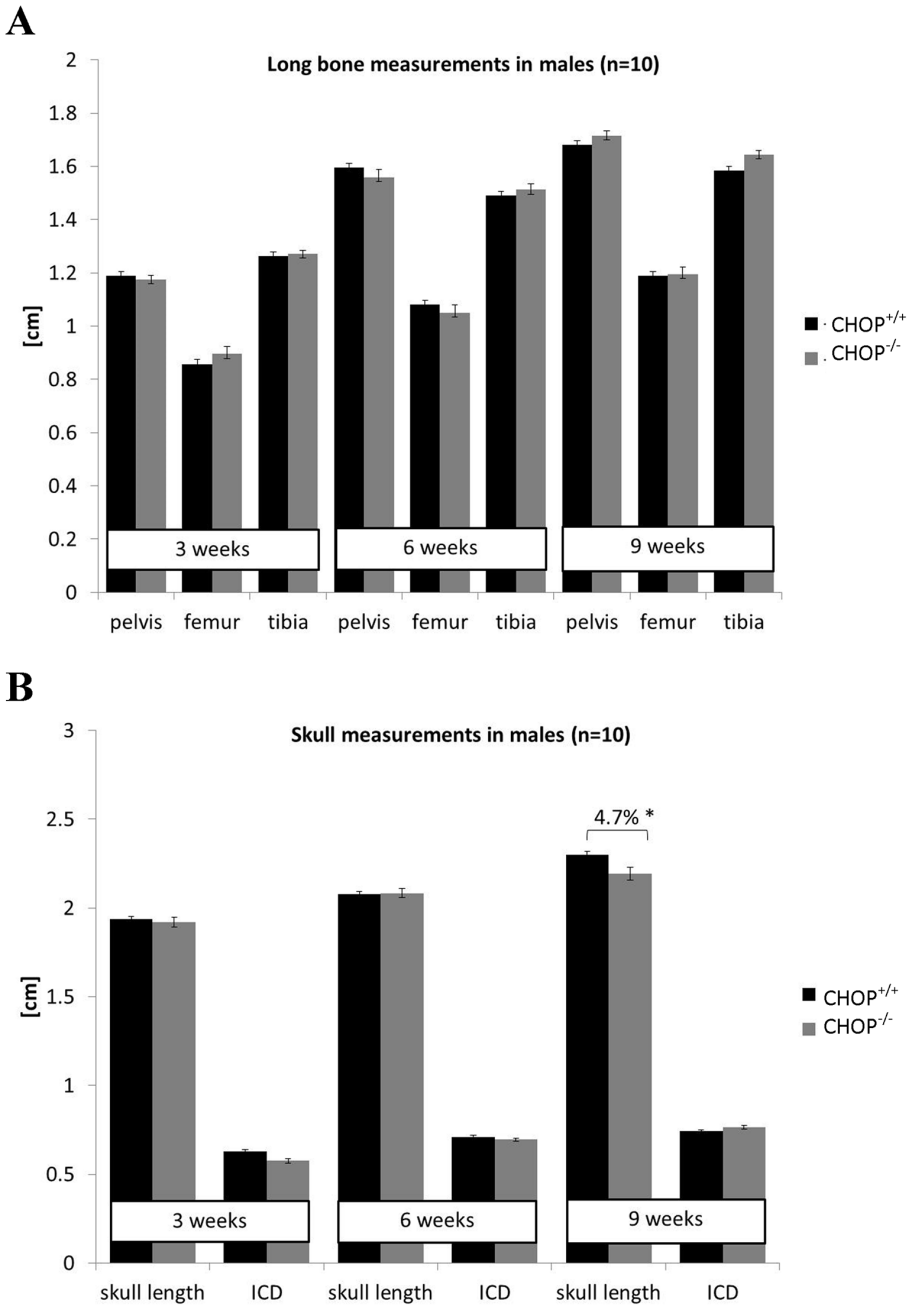
Bone measurements in mice lacking CHOP. **A**) Long bone measurements (cm) of wild type and CHOP null mice at 3, 6 and 9 weeks of age confirming that deletion of CHOP had no effect on the long bone formation (n = 10). **B**) Head measurements (cm) in wild type and CHOP null mice demonstrating shorter skulls in CHOP null mice at 9 weeks of age (n = 10; One Way ANOVA). Key: ICD = inner canthal distance; CHOP^+/+^ = wild type, CHOP^−/−^ = knock-out. Error bars show standard error of the mean (SEM); * P<0.05.

CHOP null mice were previously described as skeletally normal based on X-ray analysis performed from 1 to 12 months of age [31]. We performed a detailed analysis of radiographs from CHOP null mice at 3, 6 and 9 weeks of age and confirmed that there were no significant differences in the lengths of the tibia, pelvis and femur between wild type and CHOP null mice (Figure 1A and Table S1). Intramembraneous ossification (assessed by measuring the inner canthal distance) was also not affected by the deletion of CHOP. In contrast, the length of the skull, which is formed through a combination of endochondral and intramembranous ossification, was 4.7% shorter in CHOP null mice at 9 weeks of age when compared to the wild type controls (n = 10) (Figure 1B and Table S1). This unexpected finding suggests a role for CHOP in skull morphogenesis.

### Tibial growth plates from CHOP null mice have a regular columnar organisation of chondrocytes at 3 weeks of age and a normal ECM

**Figure 2:**
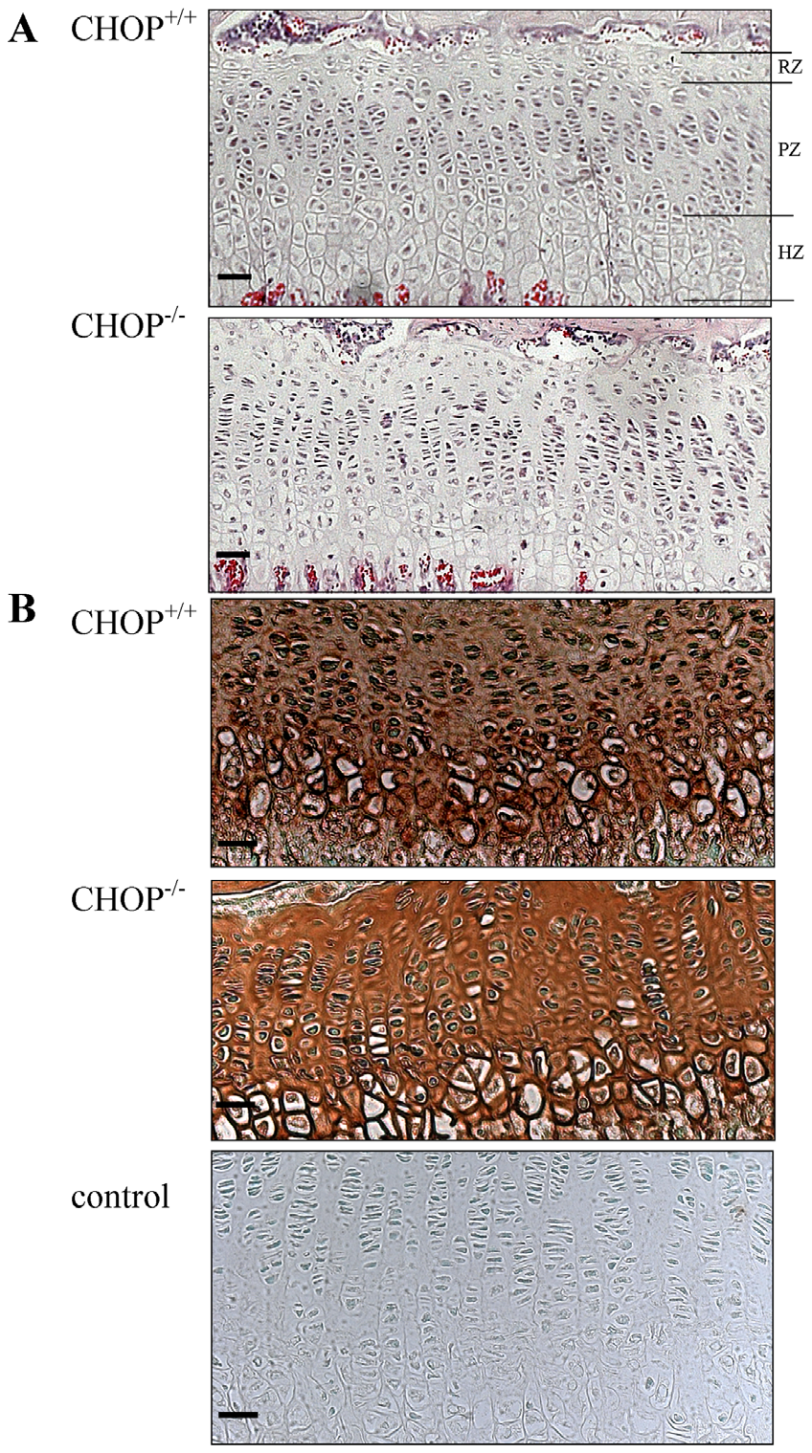
Histochemical analysis of growth plate cartilage. **A**) Haematoxylin and eosin staining of wild type and CHOP null growth plates at 3 weeks of age showing normal columnar organisation of chondrocytes in the growth plates of both genotypes and delineating the resting (RZ), proliferating (PZ) and hypertrophic (HZ) zones of the growth plate. **B**) Immunohistochemistry for COMP showing the normal distribution of COMP in wild type and CHOP null growth plate at 3 weeks of age. The negative control was generated using the secondary antibody only. Key: RZ = resting zone; PZ = proliferative zone; HZ = hypertrophic zone; CHOP^+/+^ = wild type, CHOP^−/−^ = knock-out. Scale bar for all images is 100 µm.

We analysed the morphology and composition of the tibial growth plates from 3-week-old wild type and CHOP null mice to determine the role of CHOP in endochondral ossification. H&E staining confirmed that there were no obvious differences in the overall morphology of both the wild type and CHOP null growth plates, which had regular columnar arrangements of chondrocytes in the proliferative zone with comparable thickness of the resting, proliferative and hypertrophic zones (Figure 2A, Figure S5 and not shown). The level of COMP staining was comparable between genotypes suggesting normal ECM organisation in the growth plates of CHOP null mice (Figure 2B).

### An enriched C57BL6/J genetic background increases the severity of chondrodysplasia in the T585M COMP mice

The CHOP null mice were originally generated on a C57BL6/J genetic background, whilst the background of our original T585M COMP mouse line was a combination of several different genetic strains, primarily 129Sv and CBA. Since our breeding strategy for generating double mutant mice [CHOP^−/−^/COMP^m/m^] led to an enrichment of the C57BL6/J background in the T585M COMP model, we first analysed the effect of this change in genetic background on the phenotype of the T585M COMP model prior to studying the CHOP^−/−^/COMP^m/m^ mice. To achieve this we crossed the T585M COMP mutant mice onto a C57BL6/J genetic background for 5 back-crossed generations, which resulted in approximately 97% enrichment of the C57BL6/J background in the T585M COMP mouse line [COMP^m/m^C57+].

**Figure 3:**
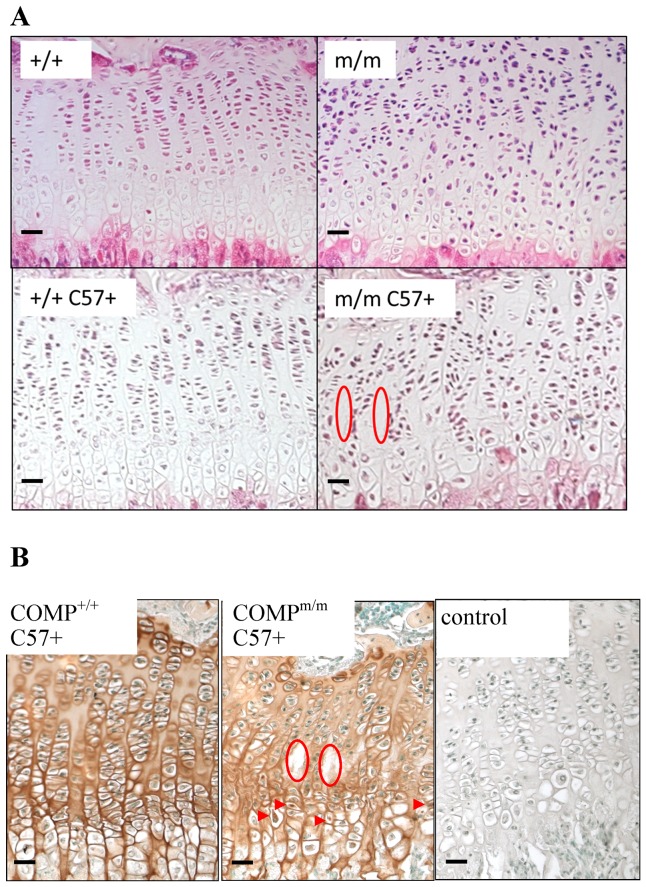
Histochemical and immunohistochemical analysis of growth plate cartilage. **A**) Haematoxylin and eosin staining of C57BL6/J enriched wild type (+/+ C57+) and T585M COMP mutant (m/m C57+) cartilage growth plates at 3 weeks of age showing further disorganisation and more hypocellular areas (red circles) in the m/m C57+ growth plates. B) Immunohistochemistry for COMP at 3 weeks of age showing abnormal columnar organisation of the growth plates in the mutant mice with areas of hypocellularity (red circles) and the retention of COMP in the T585M COMP mutant (COMP^m/m^C57+) hypertrophic zone (red arrowheads) on the C57BL6/J enriched genetic background. Positive COMP staining is brown with green nuclear counterstain. The negative control was generated using the secondary antibody only. Key: +/+ = wild type mice (mixed background); +/+ C57+ = wild type mice (enriched C57 background); m/m = T585M COMP mutant mice (mixed background); m/m C57+ = T585M COMP mutant mice (enriched C57 background). Scale bar in all images is 100 µm.

Interestingly, the growth plates of [COMP^m/m^C57+] mice were more disorganised when compared with age-matched wild type [COMP^+/+^C57+] controls and the original T585M COMP mutant mice (Figure 3A and [16]). In addition to a general disruption to the normal localisation of several ECM molecules (not shown) similar to that which has previously been reported for the T585M COMP mutant mice and manifests as reduced staining for COMP, matrilin-3 and type IX collagen in the acellular gaps between the mutant chondrocyte columns [16], we now noted that mutant COMP was retained to some extent in the chondrocytes within the hypertrophic zone of 3-week-old [COMP^m/m^C57+] mice (Figure 3B, red arrowheads).

**Figure 4:**
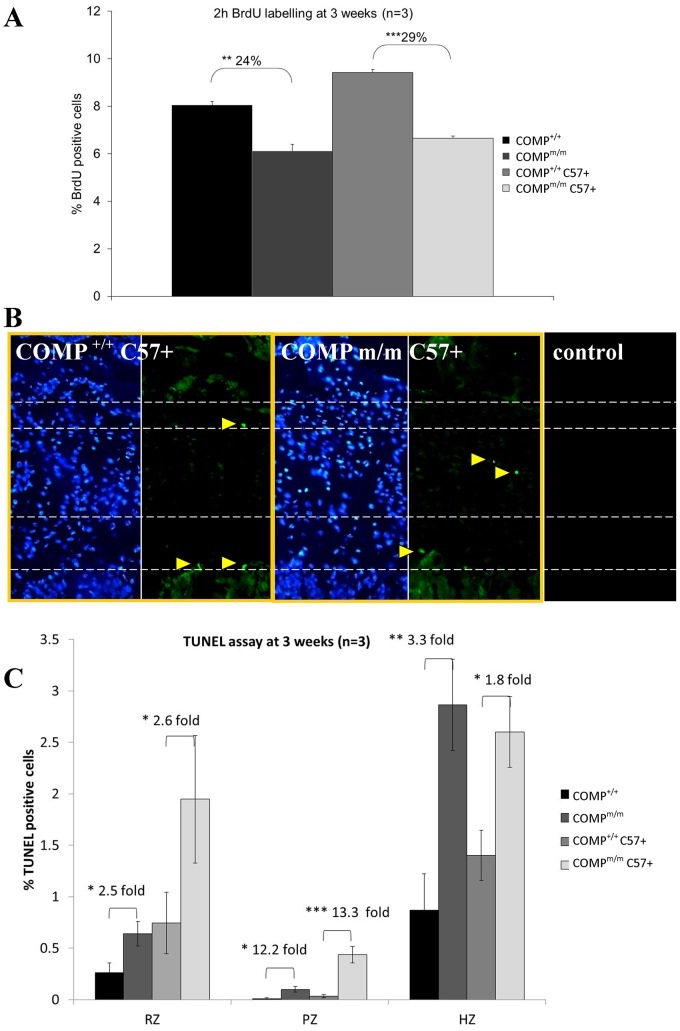
BrdU and TUNEL labelling of growth plate cartilage. **A**) The relative proportion of BrdU positive cells in wild type and T585M COMP mutant growth plates compared to the corresponding C57BL6/J enriched mice (n = 3; One Way ANOVA). This analysis demonstrated a further decrease in chondrocyte proliferation at 3 weeks of age due to the change in genetic background. **B**) Representative images of TUNEL staining in C57BL6/J enriched wild type and COMP mutant cartilage growth plates at 3 weeks of age. Green staining (yellow arrowheads) identifies TUNEL positive (apoptotic) cells against a DAPI (blue) nuclear counter stain. **C**) Quantification of the TUNEL staining in wild type and mutant growth plates at 3 weeks of age (n = 3; One Way ANOVA) showing further dysregulation of apoptosis. Key: COMP^+/+^ = wild type mice (mixed background); COMP^+/+^C57+ = wild type mice (enriched C57 background); COMP^m/m^ = T585M COMP mutant mice (mixed background); COMP^m/m^ C57+ = T585M COMP mutant mice (enriched C57 background). Error bars show standard error of the mean (SEM); * P<0.05, **P<0.01, *** P<0.001.

Furthermore, although a direct comparison between the different genetic backgrounds was not possible, we nevertheless found that chondrocyte proliferation between the mutant growth plates (and their appropriate wild type controls) was mildly decreased on the C57BL6/J background (i.e. a decrease of 24% in T585M COMP compared to 29% in [COMP^m/m^ C57+] thus representing a 17% reduction) (Figure 4A and Table S4). Finally, chondrocyte apoptosis was more dysregulated with a further increase in apoptosis in the resting and proliferating zones, but with a slight decrease in the hypertrophic zone, of growth plates from mice with an enriched C57BL6/J background (Figure 4 B and C).

### CHOP^−/−^/COMP^m/m^ mice have abnormal growth plates with more pronounced spatial disorganisation

**Figure 5:**
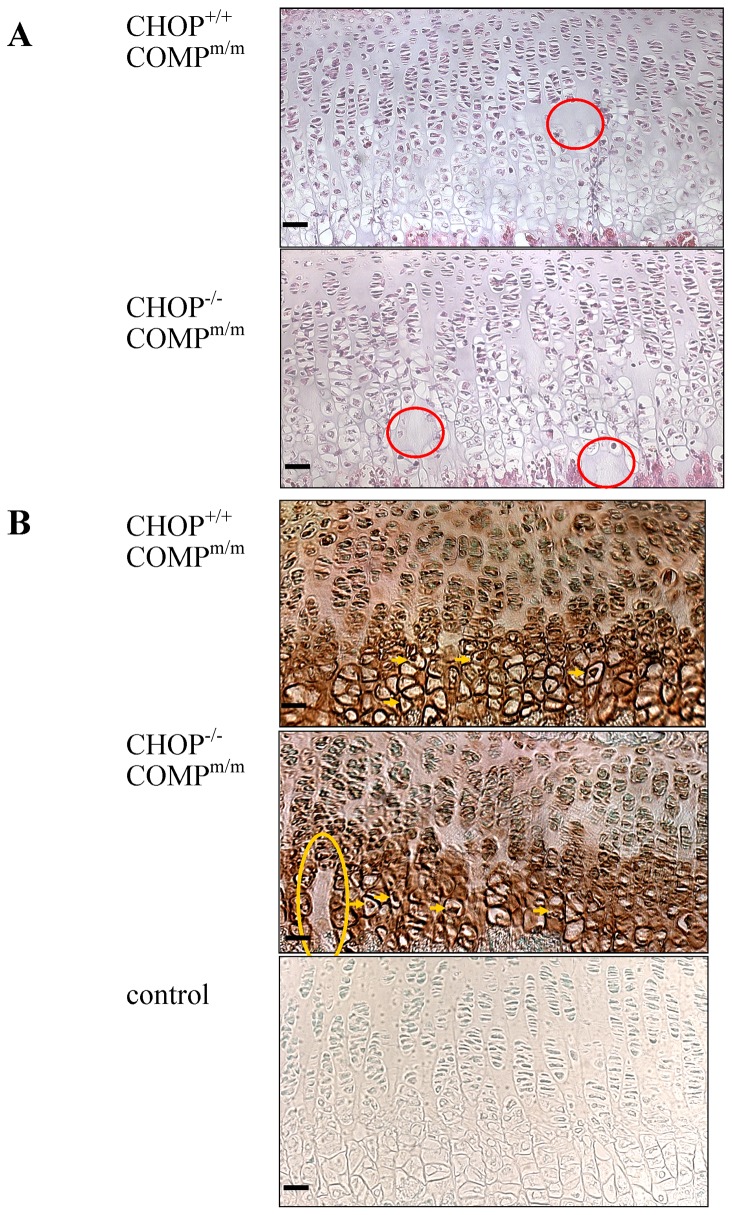
Histochemical and immunohistochemical analysis of growth plate cartilage. **A**) Haematoxylin and eosin staining of T585M COMP mutant and either CHOP wild type [CHOP^+/+^COMP^m/m^] or CHOP null [CHOP^−/−^COMP^m/m^] growth plates at 3 weeks of age. Areas of hypocellularity localised to the lower hypertrophic zone (red circles) are seen in [CHOP^−/−^COMP^m/m^] cartilage compared to [CHOP^+/+^COMP^m/m^] controls. **B**) Immunohistochemistry for COMP showing abnormal columnar organisation of the growth plate in the COMP mutant CHOP null mice [CHOP^−/−^COMP^m/m^] with areas of hypocellularity (yellow circles) in the hypertrophic zone at 3 weeks of age. Mutant COMP was also retained in chondrocytes of the hypertrophic zone of both [CHOP^+/+^COMP^m/m^] and [CHOP^−/−^COMP^m/m^] due to the enriched C57BL6/J background in this model (see yellow arrows). Positive staining for COMP is brown with a green nuclear counterstain. The negative control was generated using a secondary antibody only. Key: CHOP^+/+^COMP^m/m^ = CHOP wild type and T585M COMP mutation (homozygous); CHOP^−/−^COMP^m/m^ = CHOP null and T585M COMP mutation (homozygous). Scale bar in all images is 100 µm.

To determine the role of CHOP in the pathobiology of CTD-COMP-related PSACH, we crossed the T585M COMP mice with the CHOP null mice and analysed the resulting phenotype. By 3 weeks of age the tibial growth plates of [CHOP^−/−^/COMP^m/m^] mice appeared further disorganised compared to [CHOP^+/+^/COMP^m/m^] mice with more pronounced spaces between the columns of chondrocytes within the proliferative zone, and also a distinct hypocellular appearance to the hypertrophic zone (Figure 5A). This disorganisation to the hypertrophic zone had not been previously observed in the T585M COMP mutant mice, or in the [COMP^m/m^ C57+] mice, and therefore appeared to be the direct result of CHOP ablation.

### The organisation of the extracellular matrix is not affected by CHOP ablation in T585M COMP mutant mice

Immunohistochemistry for COMP, matrilin-3 and type IX collagen was performed on the cartilage growth plates from 3 week old [CHOP^−/−^/COMP^m/m^] and [CHOP^+/+^/COMP^m/m^] mice (Figure 5B and not shown). COMP staining in the ECM was depleted in a similar fashion to that previously reported for the T585M COMP mice [16]. Moreover, a proportion of mutant COMP was retained within the chondrocytes of the hypertrophic zone of both [CHOP^−/−^/COMP^m/m^] and [CHOP^+/+^/COMP^m/m^] mice (Figure 5B), which may be directly due to the enriched C57BL6/J genetic background, rather than deletion of CHOP itself. Staining for matrilin-3 and type IX collagen followed the same distribution as mutant COMP (not shown) and there appeared to be no differences in the distribution of these ECM components between the [CHOP^−/−^/COMP^m/m^] and [CHOP^+/+^/COMP^m/m^] growth plate cartilages.

### The deletion of CHOP alleviates abnormal chondrocyte apoptosis in the resting zone of T585M COMP mutant growth plates but does not affect proliferation

**Figure 6:**
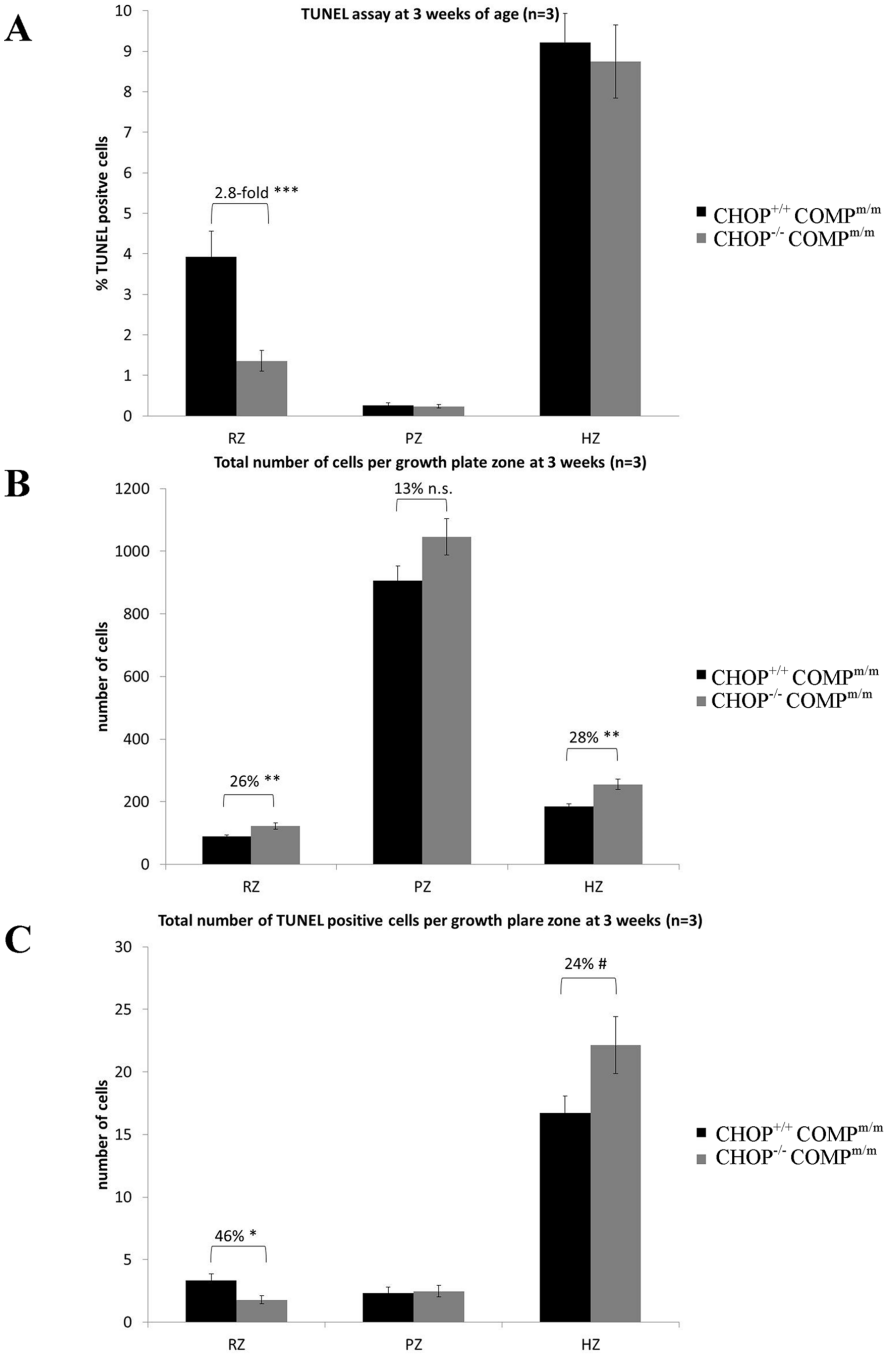
TUNEL assay and cell quantification in growth plate at 3 weeks of age. **A**) Quantification of the TUNEL staining in T585M COMP mutant and either CHOP wild type [CHOP^+/+^COMP^m/m^] or CHOP null [CHOP^−/−^COMP^m/m^] growth plates. This analysis shows a significant decrease in apoptosis in the resting zone following deletion of CHOP (n = 3; One Way ANOVA). **B)** The average number of cells per growth plate zone showing a significant increase in the RZ and HZ and a trend towards an increase in the PZ following deletion of CHOP (n = 3; One Way ANOVA). **C**) The average number of TUNEL positive cells per growth plate zone showing a significant decrease in the RZ and a trend towards an increase in the HZ of the [CHOP^−/−^COMP^m/m^] growth plates (n = 3; One Way ANOVA). Key: CHOP^+/+^COMP^m/m^ = CHOP wild type and T585M COMP mutation (homozygous); CHOP^−/−^COMP^m/m^ = CHOP null and T585M COMP mutation (homozygous); RZ = resting zone; PZ = proliferative zone; HZ = hypertrophic zone. Error bars show standard error of the mean (SEM); # P = 0.06, * P<0.05, ** P<0.01, *** P<0.001.

**Figure 7:**
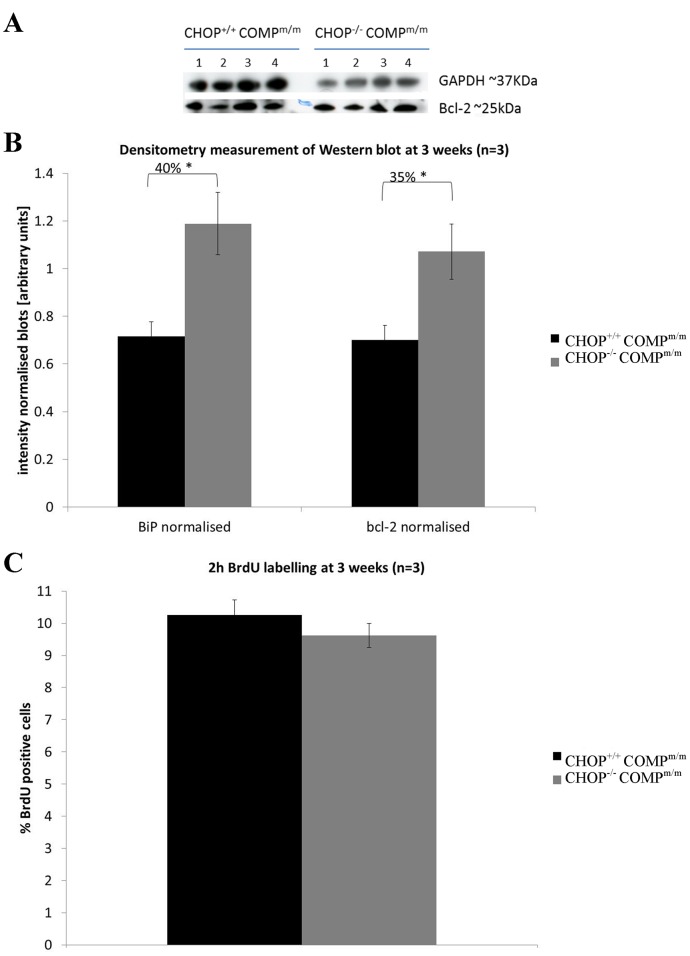
Western blot and BrdU analysis of mouse cartilage at 3 weeks of age. **A**) A representative image of a bcl-2 Western blot on mouse xiphoid homogenates at 3 weeks. 4 mice per genotype (1–4); GAPDH was used as a loading control. **B**) Densitometry of Western blots of cartilage homogenates from xiphoid cartilage showing an significant increase in bcl-2 and BiP protein after CHOP removal (n = 3; t-test) **B**) Quantification of the relative number of BrdU positive cells in T585M COMP mutant and either CHOP wild type [CHOP^+/+^COMP^m/m^] or knock-out [CHOP^−/−^COMP^m/m^] growth plate showing that CHOP deletion had no effect on chondrocyte proliferation (n = 3; t-test). Key: CHOP^+/+^COMP^m/m^ = CHOP wild type and T585M COMP mutation (homozygous); CHOP^−/−^COMP^m/m^ = CHOP null and T585M COMP mutation (homozygous). Error bars show standard error of the mean (SEM); * P<0.05.

TUNEL assay was performed on the growth plates of 3-week-old mice to determine whether the deletion of CHOP would ablate the abnormal chondrocyte apoptosis observed in the T585M COMP mutant growth plates. At 3 weeks of age the relative levels of apoptosis in the resting zone of [CHOP^−/−^/COMP^m/m^] mice were decreased by 2.8-fold (p<0.001) when compared to [CHOP^+/+^/COMP^m/m^] mice. However, apoptosis was not improved in the proliferative or hypertrophic zones of the growth plate (Figure 6A and Table S4). Furthermore, the decrease in apoptosis in the resting zone of [CHOP^−/−^/COMP^m/m^] mice resulted in a trend towards a greater number of chondrocytes in each growth plate zone (Figure 6B; 26% p<0.01 in the resting zone; 13% p = 0.08 (n.s.) in the proliferative zone; 28% p<0.01 in the hypertrophic zone). The increased number of cells per zone did not have an impact on the growth plate thickness at 3 weeks of age (Figure S5). We also noticed a trend towards a higher absolute number of TUNEL positive cells in the terminal hypertrophic zone of [CHOP^−/−^/COMP^m/m^] mice (Figure 6C; 22% p = 0.06). The relative level of the important anti-apoptotic protein bcl-2 was increased by 35% in [CHOP^−/−^/COMP^m/m^] mice (Figure 7A and B), which may reflect the decrease in apoptosis observed in the resting zone of [CHOP^−/−^/COMP^m/m^] mice. In addition the relative level of the key UPR chaperone protein BiP was increased by 40% following the deletion of CHOP in T585M COMP mice (Figure 7B and Table S3).

BrdU labelling was performed at 3 weeks of age to determine the relative levels of chondrocyte proliferation in the growth plates of [CHOP^−/−^/COMP^m/m^] and [CHOP^+/+^/COMP^m/m^] mice. Chondrocyte proliferation was comparable between the two genotypes (Figure 7C and Table S4; 10.25%±0.47 and 9.62%±0.38 respectively) indicating that CHOP signalling had no effect on the abnormal proliferation levels in the original T585M COMP mice.

### Deletion of CHOP leads to a decrease in the levels of several bone maturation markers

Mass spectrometry was performed on the proteins extracted from homogenized 3 week old xiphoid cartilage from T585M COMP mutant mice either wild type or null for CHOP (Table 1). Relative total amounts of protein at the start of the experiment were taken into account and spectral counting showed significant changes in the levels of several proteins involved in bone homeostasis and maturation including decorin, perilipin and thrombospondin-1. Fibrillin, a protein important for osteoblast maturation and fibromodulin and HPLN1, both of which are expressed in the hypertrophic zone of the cartilage and in bone, were also decreased in the xiphoid of [CHOP^−/−^/COMP^m/m^] cartilage. Finally, we detected a decrease in the relative levels of nebulin, which may also be involved in cartilage/bone biology.

**Table 1:** Mass spectrometry (LC-MS/MS) of T585M mutant and CHOP wild type [CHOP^+/+^/COMP^m/m^] or knock-out [CHOP^−/−^/COMP^m/m^] cartilage at 3 weeks of age.

gene	protein	Average spectra number [CHOP^+/+^/COMP^m/m^]	Average spectra number [CHOP^−/−^/COMP^m/m^]	P value
chad	chondroadherin	2	0	0.004 **
fmod	fibromodulin	2	0	0.006 **
dcn	decorin	3.25	0	0.007 **
fbn1	fibrillin-1	10	2.75	0.02 *
tsp1	thrombospondin-1	0	1.25	0.03 *
neb	nebulin-1	13.25	6.25	0.04 *
hpln1	hyaluronan and proteoglycan link protein 1	2.5	0.5	0.05 *
plin1	perilipin-1	1.75	0	0.05 *

The average number of spectra are shown for each genotypes (n = 4).

### The long bones and skulls are shorter in [CHOP^−/−^/COMP^m/m^] mice

**Figure 8:**
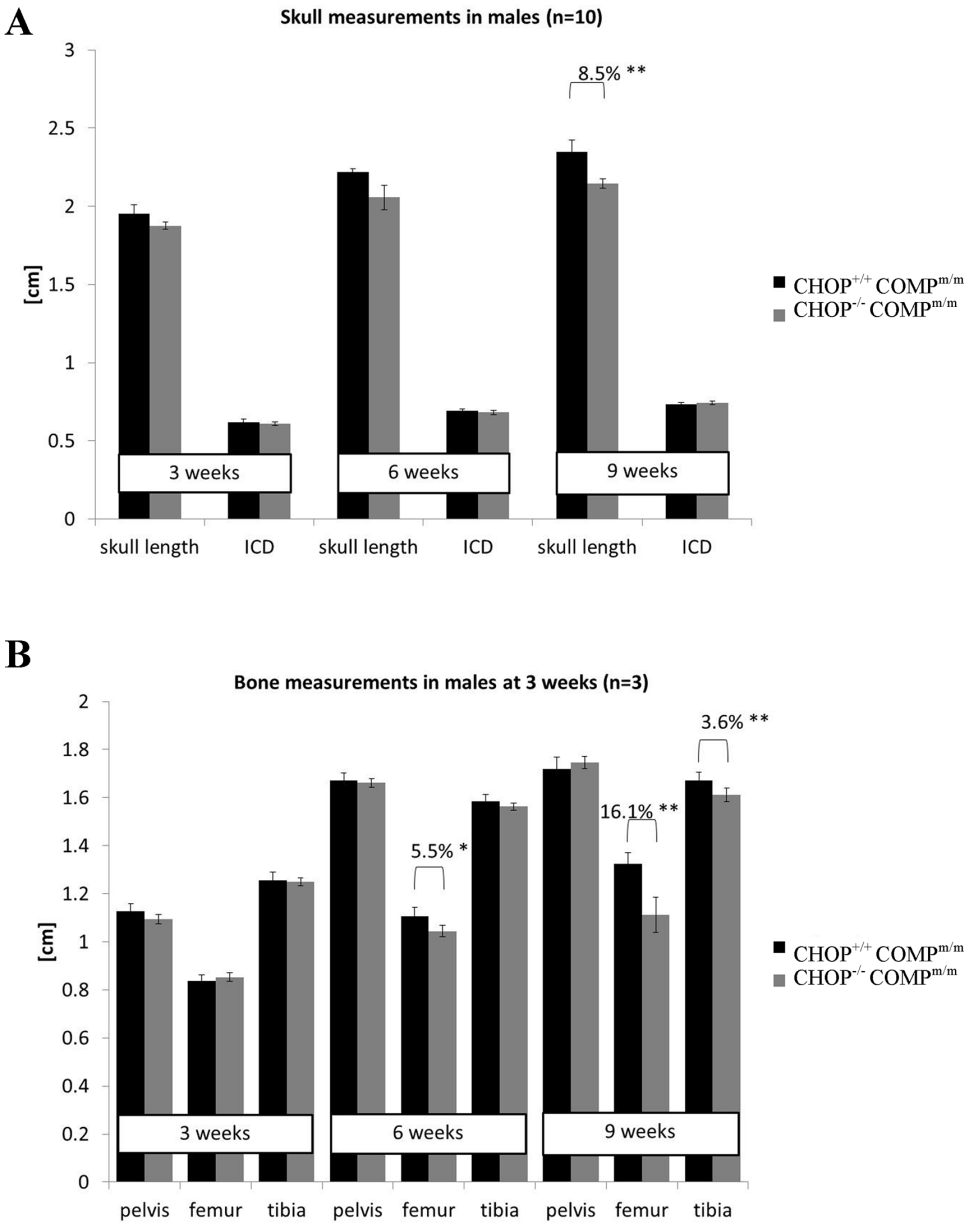
Head and bone measurements in COMP mutant and CHOP null mice. **A**) Head measurements in T585M COMP mutant and either CHOP wild type [CHOP^+/+^COMP^m/m^] or CHOP null [CHOP^−/−^COMP^m/m^] showing shorter skulls in [CHOP^−/−^COMP^m/m^] at 9 weeks of age (n = 10; One Way ANOVA). **B**) Long bone measurements in [CHOP^−/−^COMP^m/m^] mice showing a further exacerbated short limbed dwarfism following CHOP deletion (n = 10; One Way ANOVA). Error bars represent standard error of the mean (SEM) Key: Key: CHOP^+/+^COMP^m/m^ = CHOP wild type and T585M COMP mutation (homozygous); CHOP^−/−^COMP^m/m^ = CHOP null and T585M COMP mutation (homozygous); ICD = inner canthal distance. Error bars represent standard error of the mean (SEM); * P<0.05, ** P<0.01, *** P<0.001.

[CHOP^−/−^/COMP^m/m^] and [CHOP^+/+^/COMP^m/m^] mice were radiographed at 3, 6 and 9 weeks of age to determine the effect of CHOP ablation on bone growth in T585M COMP mutant mice. At 6 weeks of age the femurs in [CHOP^−/−^/COMP^m/m^] mice were 5.5% shorter than in the control mice and by 9 weeks this difference had reached 15.4% (Figure 8B and Table S2; p<0.05 and p<0.001 respectively). In addition, by 9 weeks of age the skulls (8% p<0.05) and tibia (3.8% p<0.01) were also shorter in [CHOP^−/−^/COMP^m/m^] mice (Figure 8A and B), suggesting a potential synergistic effect of the T585M COMP mutation and CHOP ablation on bone growth. Interestingly a hip dysplasia [16], measured by the angle between the ischial and the pubic bone, was alleviated by the deletion of CHOP (6 weeks, 25% decrease p<0.01; 9 weeks 8% decrease p<0.05; data not shown).

## Discussion

Pseudoachondroplasia (PSACH) is characterised by short-limbed dwarfism, a waddling gait and early onset osteoarthritis [3], [4]. PSACH is believed to result exclusively from mutations in two distinct domains of cartilage oligomeric matrix protein (COMP); the thrombospondin type 3 repeat region (T3) and the C-terminal globular domain (CTD) [1], [32], [33]. *In vitro* analysis of cartilage from PSACH patients with a range of different T3-COMP mutations consistently shows enlarged rER that can have a characteristic lamellar appearance. Following these observations it was hypothesised that PSACH resulted from an unfolded protein response (UPR) elicited by misfolded COMP retained in the ER of chondrocytes [34], [35].

In contrast, the pathology of CTD-COMP mutations appeared more complex and it was demonstrated in several studies that these mutations often allow the folding and secretion of the mutant protein [15], [36]. Nevertheless, in a gene targeted mouse model of a specific CTD-COMP mutation (T585M), the expression and secretion of this mutant protein resulted in dysregulated chondrocyte apoptosis and decreased proliferation. Moreover, the relative levels of the anti-apoptotic protein bcl-2 were decreased and mild ER stress was detected in mutant chondrocytes, including an increase in the levels of the ER stress related and pro-apoptotic transcription factor CHOP [16]. We therefore considered that CHOP might be an important factor in the pathogenesis of T585M-COMP induced PSACH and that the dysregulated chondrocyte apoptosis was induced by ER stress and mediated by CHOP. We hypothesised that crossing the T585M-COMP mice onto CHOP null genetic background [22] would alleviate the PSACH phenotype.

To determine the role of CHOP in T585M-COMP pathology we crossed the T585M-COMP mice onto a CHOP null background, but during this process we enriched the C57BL6/J background of the original T585M-COMP mice. Genetic background is an important consideration when performing studies on animal models of disease and breeding mice onto various defined strains can dramatically increase or decrease disease severity [27], [37]. In the first instance we therefore analysed what effect such change in the genetic background would have on the phenotypic severity in the T585M-COMP model.

We found that the PSACH phenotype on the enriched C57BL6/J background was slightly more severe. In particular, the columns of chondrocytes in the proliferative zone were further disrupted and mutant COMP was also visibly retained in the hypertrophic chondrocytes of the cartilage growth plates, thereby resembling the more severe T3-COMP pathology as previously reported [14]. Moreover, at 3 weeks of age the chondrocyte apoptosis was slightly increased and proliferation was further decreased, suggesting the influence of a background-specific genetic modifier of disease severity.

CHOP null mice have previously been described as skeletally normal based on full body radiographs taken between 1 and 12 months of age [31]. They have normal osteoblast numbers but show decreased rates of trabecular bone formation and it was hypothesised that CHOP is important for normal osteoblast function. Furthermore, the overexpression of CHOP in an *in vitro* system favours differentiation of stromal cells towards osteoblasts, indicating an important role for CHOP in bone biology [38]. In addition, the levels of osteocalcin and type I collagen expression were decreased in the calvarial bones from 3 month old CHOP null mice [31]. In this study we confirm that CHOP null mice are overall skeletally normal and that neither endochondral nor intramembranous ossification appears to be disrupted. However, CHOP does appear to play a role in skull formation, as evidenced by our X-ray measurements showing 4.7% shorter skulls in CHOP null mice at 9 weeks of age.

CHOP null mice have correctly organised cartilage growth plates with a typical columnar arrangement of chondrocytes within the proliferative zone. In contrast, T585M-COMP mutant mice present with disruption to the columnar organisation characterised by rounded clusters of cells and spaces between the individual columns of chondrocytes, particularly in the proliferative zone [16]. This phenotype appeared further exacerbated following the deletion of CHOP such that the spaces extended towards the hypertrophic zone and the vascular invasion front in the growth plates of [CHOP^−/−^/COMP^m/m^] mice. The secretion of key extracellular matrix proteins appeared similar between genotypes suggesting that no additional disruptions to the ECM organisation were induced by the deletion of CHOP. A slight retention of mutant T585M-COMP in chondrocytes of the hypertrophic zone was noted and this was not alleviated by the deletion of CHOP.

Chondrocyte proliferation was not affected in [CHOP^−/−^/COMP^m/m^] mice and this is in agreement with previous studies on both CHOP-null mice and mice overexpressing CHOP in bone, both of which did not show any difference in BrdU incorporation in osteoblasts and suggesting that CHOP *per se* does not play a significant role in cell proliferation [31], [38]. Chondrocyte apoptosis was only decreased in the resting zone of [CHOP^−/−^/COMP^m/m^] mice suggesting that abnormal chondrocyte apoptosis in the proliferative zone, which is hypothesised to be one of the key factors in the disease aetiology of T585M-COMP related PSACH, is in fact not CHOP mediated. Interestingly, the decrease in apoptosis in the resting zone resulted in an absolute increase in the total number of cells in the proliferative and hypertrophic zones, and by inference, an increase in the overall number of cells undergoing apoptosis in the hypertrophic zone. The exacerbated growth plate pathology may therefore be a direct result of this additional dysregulation of chondrocyte apoptosis.

Interestingly, a mild increase in CHOP was also detected in a tetracycline-inducible transgenic mouse model of T3-COMP PSACH [39], whereby mutant COMP protein was retained within the ER of chondrocytes leading to an increase in apoptosis and a short-limbed dwarfism. Furthermore, anti-apoptotic transcripts (such as bcl-2 and bag3) were decreased, whereas pro-apoptotic genes (including CHOP) were mildly increased in the mutant growth plates, along with other oxidative stress and DNA damage markers [39]. When CHOP was deleted in these transgenic mice, mutant protein retention was alleviated, abnormal apoptosis was decreased and chondrocyte proliferation increased. Moreover, the authors noted a decrease in the levels of other ER stress inducible genes suggesting a potential role of CHOP in PSACH pathogenesis [40]. However, it is important to note that transgenic models of disease, although appropriate for modelling certain molecular interactions, often result in over-expression of the mutant protein and therefore do not always accurately model the disease pathology. Transcriptomic analysis of the more physiologically relevant knock-in mouse model of T3-COMP PSACH showed no increased expression of ER stress related genes including CHOP and suggested that T3-COMP PSACH results from a combination of oxidative stress, apoptosis and cell cycle arrest [14].

CHOP is an early marker of stress in response to ER overload and its expression differs between acute and mild, but prolonged, UPR [18], [41]. Furthermore, it has been recently shown that CHOP mRNA transcripts and indeed CHOP protein itself have short half lives in prolonged mild ER stress [41]. It is therefore interesting to speculate that CHOP-mediated cell death in the resting zone of [CHOP^−/−^/COMP^m/m^] mice is an initial selection mechanism against the ER stress induced by mutant COMP expression whereby the cells that cannot resolve the processing of mutant protein die via ER-stress mediated apoptosis, whilst the remainder continue to proliferate and differentiate as normal. Conversely, in the transgenic model the overexpression of COMP may result in ER overload and a higher more prolonged upregulation of CHOP.

CHOP is known to signal directly to downregulate the major anti-apoptotic protein bcl-2 [21]. In [CHOP^−/−^/COMP^m/m^] mice the bcl-2 protein levels in cartilage were increased by 40%, which may reflect the positive effect that the deletion of CHOP had on apoptosis of chondrocytes in the resting zone. Moreover, BiP levels were significantly increased in T585M-COMP mutant chondrocytes following the deletion of CHOP. The induction of glucose-regulated proteins including BiP (also known as Grp78) during the mild prolonged ER stress is a late event compared to CHOP induction [18]. Therefore, an increase in the relative levels of BiP may be perceived as a marker of mild persistent ER stress in [CHOP^−/−^/COMP^m/m^] chondrocytes and could be identifying the population of resting chondrocytes which have not died via ER-stress mediated apoptosis. An increase in BiP is thought to inhibit the progression of late ER stress signalling events since more chaperone molecules are synthesised to aid in folding of the mutant protein [18]. Plasma cells, which are a good example of ‘professional’ secretory cells, are known to customise their UPR during differentiation and in preparation for the secretion of immunoglobulins [41]. They adapt to the persistent mild ER stress via a stable up-regulation of BiP and other molecular chaperones [42]. Growth plate chondrocytes also secrete large amounts of large complex extracellular matrix proteins; it is therefore interesting to speculate that they might also possess a similar ability to adapt to physiological and pathological stresses.

The further dysregulation of the cartilage growth plates of [CHOP^−/−^/COMP^m/m^] mice may contribute to the overall decrease in long bone length and exacerbation of the PSACH phenotype. Moreover, the skulls of [CHOP^−/−^/COMP^m/m^] mice were also shorter than those of T585M-COMP mice suggesting a potential synergistic effect of mutant COMP and CHOP ablation in bone formation. The role of CHOP in skull morphogenesis, although beyond the scope of this study, is an interesting topic and will be investigated further.

Overall these data suggest that the synergy between the C-terminal COMP mutation (T585M) and a deletion of CHOP, which leads to the exacerbated PSACH phenotype, is most likely manifested at the bone/cartilage interphase. This is possibly due to role of CHOP in osteoblast differentiation, which combined with the abnormal pre-ossification cartilage template arising through mutant COMP deposition in the cartilage ECM could in turn influence the differentiation of osteoblasts. Moreover, mass spectrometry of the xiphoid cartilage from 3 week old mice revealed a decrease in the levels of proteins important for bone maturation, in COMP mutant CHOP null mice when compared with the COMP mutant mice alone.

The deletion of CHOP in the T585M-COMP model did not affect the relative levels of apoptosis in the proliferative and hypertrophic zones of the mutant growth plates, suggesting a mechanism of cell death that was not CHOP-mediated. We have previously shown that the ECM in the original T585M-COMP cartilage is noticeably disrupted in the proliferative and hypertrophic zones, whilst cell morphology is also abnormal. We therefore hypothesise that chondrocyte apoptosis in these zones is a consequence of the abnormal extracellular environment, rather than due to the processing of the mutant protein *per se*, and as such may be mediated by different genetic pathways.

In summary, we have shown that CHOP plays an important role in an early stress response in T585M-COMP related PSACH and further confirmed a role for CHOP in bone pathobiology. We have also shown that in the absence of CHOP the chondrocytes can adapt during mild ER stress and that the mechanism underlying abnormal chondrocyte apoptosis in the proliferative zone of T585M-COMP PSACH is most likely due to an extracellular stress induced by the abnormal architecture of the mutant ECM. Our study therefore provides a new insight into the role of mild ER stress in PSACH disease progression and excludes CHOP as a potential therapeutic target in the CTD-COMP forms of PSACH.

## Supporting Information

Figure S1 **Full length x-ray image of a mouse homozygous for T585M mutant COMP (m/m) showing the five bone length measurements used in this study.**
(TIF)

Figure S2 **A**) Representative image of BrdU immunostaining following the 2 h BrdU labelling of the cartilage growth plate in 3 week old mice. The black lines delineate the proliferative zone where the BrdU positive cells were counted individually and expressed as a percentage of all the cells in the zone. **B**) Negative (secondary only) control. Scale bar is 100 µm.
(TIF)

Figure S3 **A**) Haematoxylin and eosin staining of a wild type mouse xiphoid process from a 3 week old mice showing both the bone and also growth plate cartilage. **B**) Enlarged xiphoid growth plate section showing easily recognisable resting, proliferative and hypertrophic zones. Key: HZ = hypertrophic zone, PZ = proliferative zone, RZ = resting zone. Scale bar 200 µm.
(TIF)

Figure S4 **A**) Immunostaining of a wild type mouse xiphoid process from a 3 week old mice for the cartilage markers COMP, matrilin-3 and type IX collagen confirming the cartilaginous tissue in the xiphoid process. **B**) Immunostaining for type X collagen (a marker of hypertrophic chondrocytes) and osteopontin (OPN; a bone and skeletal muscle marker) in the xiphoid process at 3 weeks of age. In all images positive staining is brown with a green nuclear counterstain and the negative control is the secondary antibody only. Scale bar is 200 µm.
(TIF)

Figure S5 **Measurement of the growth plate thickness from the vascular invasion front to the top of the resting zone in [COMP m/m CHOP +/+] and [COMP m/m CHOP −/−] growth plates at 3 weeks of age (n = 5; t-test).**
(TIF)

Table S1 **Raw data for bone measurements in CHOP wild type and CHOP null mice (n = 10, One Way ANOVA).** Standard error of the mean. Key: ICD inner canthal distance, +/+ wild type, −/− knockout (null). * P<0.05.
(DOCX)

Table S2 **Raw data for bone measurements in COMP m/m CHOP +/+ and COMP m/m CHOP −/− mice (n = 10, One Way ANOVA).** Standard error of the mean. Key: ICD inner canthal distance, +/+ wild type, −/− knockout (null), m/m homozygous mutant. * P<0.05, ** P<0.01.
(DOCX)

Table S3 **Densitometry measurement of COMP m/m CHOP +/+ and CHOP −/− Western blots at 3 weeks (n = 4, t-test).** Standard error of the mean. Key: +/+ wild type, −/− knockout (null), m/m homozygous mutant. * P<0.05.
(DOCX)

Table S4 **Raw data for BrdU analysis and TUNEL analysis included in the paper (n = 3, One Way ANOVA).** Standard error of the mean. Key: RZ resting zone, PZ proliferative zone, HZ hypertrophic zone, +/+ wild type, −/− knockout (null), m/m homozygous mutant. * P<0.05, ** P<0.01, *** P<0.001.
(DOCX)
