# Supplementary figures and images for: Abnormal Chondrocyte Apoptosis in the Cartilage Growth Plate is Influenced by Genetic Background and Deletion of CHOP in a Targeted Mouse Model of Pseudoachondroplasia
Source: PLoS One. 2014 Feb 18;9(2):e85145. doi: 10.1371/journal.pone.0085145 (PMC3928032; doi:10.1371/journal.pone.0085145)

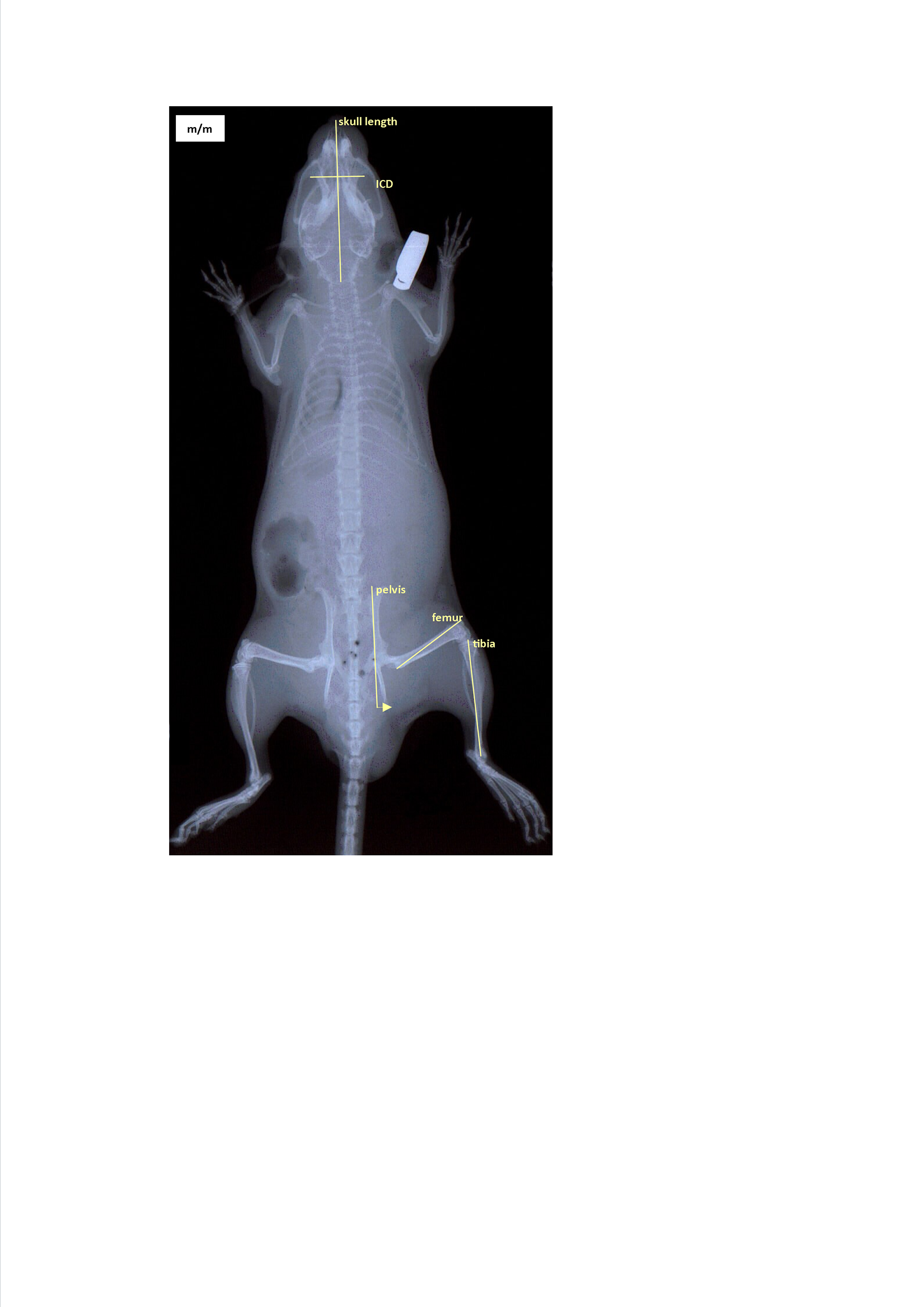

Supplement: Figure S1 — Full length x-ray image of a mouse homozygous for T585M mutant COMP (m/m) showing the five bone length measurements used in this study. (TIF) [file pone.0085145.s001.tif]

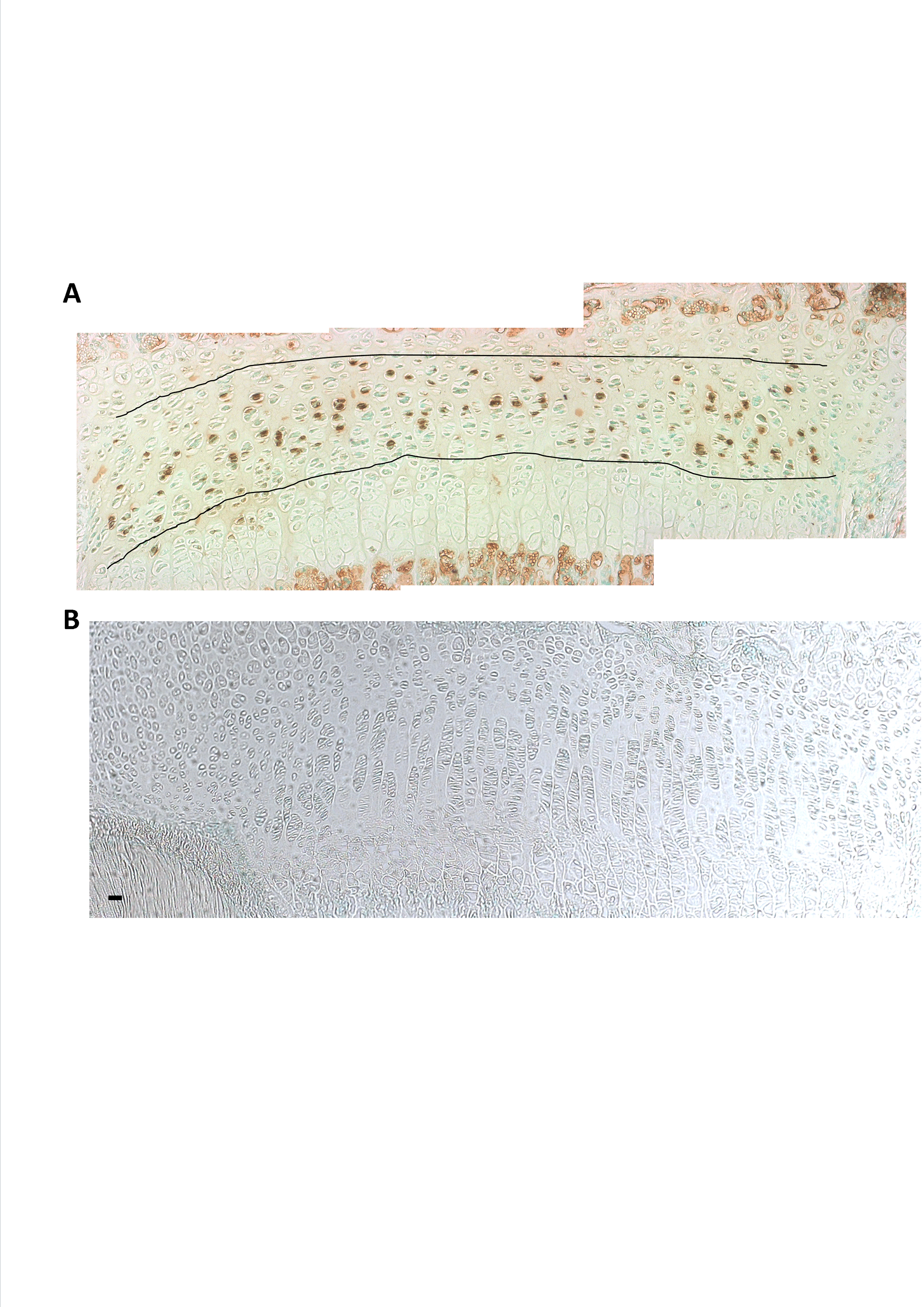

Supplement: Figure S2 — A) Representative image of BrdU immunostaining following the 2 h BrdU labelling of the cartilage growth plate in 3 week old mice. The black lines delineate the proliferative zone where the BrdU positive cells were counted individually and expressed as a percentage of all the cells in the zone. B) Negative (secondary only) control. Scale bar is 100 µm. (TIF) [file pone.0085145.s002.tif]

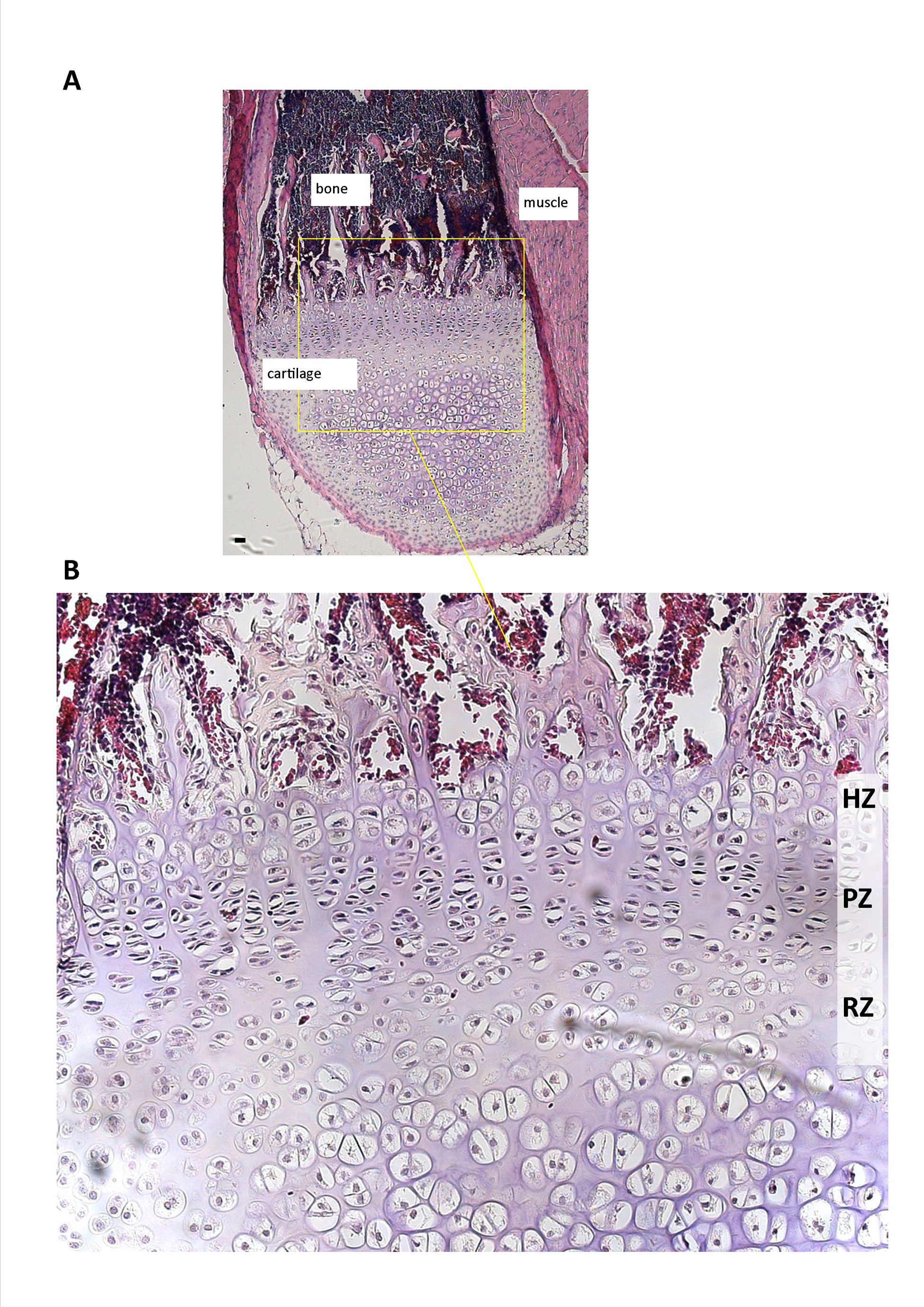

Supplement: Figure S3 — A) Haematoxylin and eosin staining of a wild type mouse xiphoid process from a 3 week old mice showing both the bone and also growth plate cartilage. B) Enlarged xiphoid growth plate section showing easily recognisable resting, proliferative and hypertrophic zones. Key: HZ = hypertrophic zone, PZ = proliferative zone, RZ = resting zone. Scale bar 200 µm. (TIF) [file pone.0085145.s003.tif]

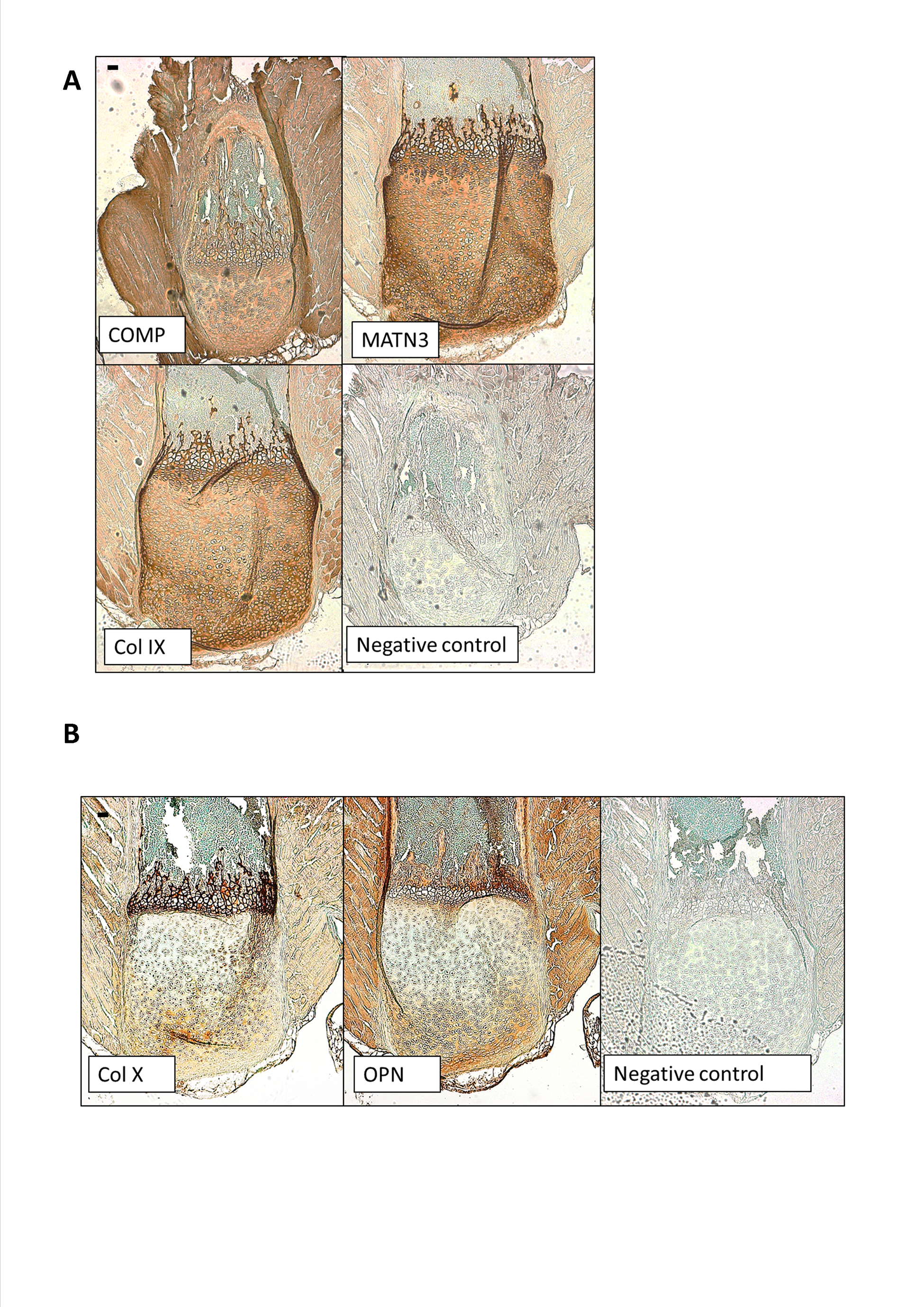

Supplement: Figure S4 — A) Immunostaining of a wild type mouse xiphoid process from a 3 week old mice for the cartilage markers COMP, matrilin-3 and type IX collagen confirming the cartilaginous tissue in the xiphoid process. B) Immunostaining for type X collagen (a marker of hypertrophic chondrocytes) and osteopontin (OPN; a bone and skeletal muscle marker) in the xiphoid process at 3 weeks of age. In all images positive staining is brown with a green nuclear counterstain and the negative control is the secondary antibody only. Scale bar is 200 µm. (TIF) [file pone.0085145.s004.tif]

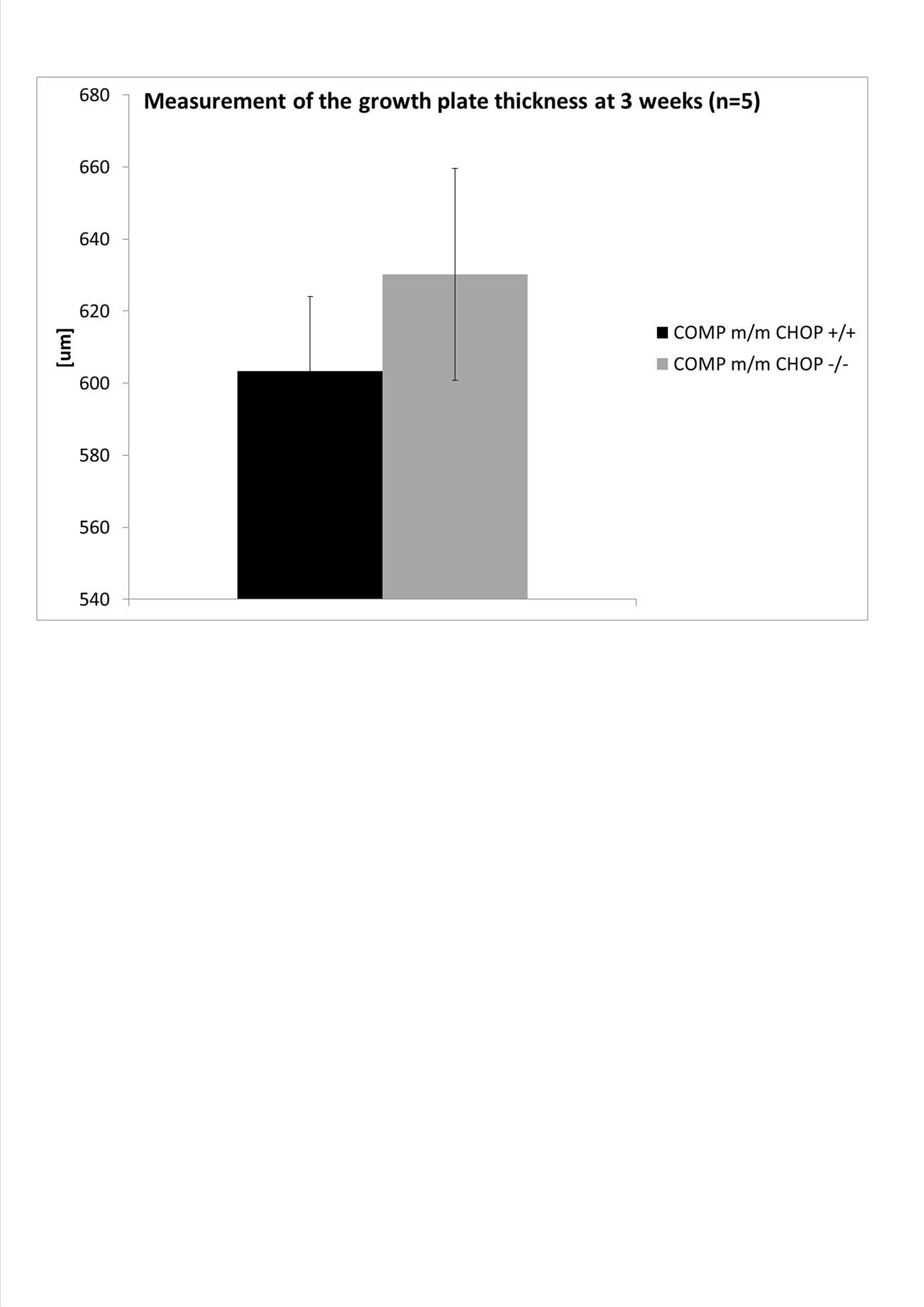

Supplement: Figure S5 — Measurement of the growth plate thickness from the vascular invasion front to the top of the resting zone in [COMP m/m CHOP +/+] and [COMP m/m CHOP −/−] growth plates at 3 weeks of age (n = 5; t-test). (TIF) [file pone.0085145.s005.tif]
