# Supplementary material for: Abnormal Chondrocyte Apoptosis in the Cartilage Growth Plate is Influenced by Genetic Background and Deletion of CHOP in a Targeted Mouse Model of Pseudoachondroplasia
Source: PLoS One. 2014 Feb 18;9(2):e85145. doi: 10.1371/journal.pone.0085145 (PMC3928032; doi:10.1371/journal.pone.0085145)
Supplement: Table S1 — Raw data for bone measurements in CHOP wild type and CHOP null mice (n = 10, One Way ANOVA). Standard error of the mean. Key: ICD inner canthal distance, +/+ wild type, −/− knockout (null). * P<0.05. (DOCX) [file pone.0085145.s006.docx]

| **Table S1** | |  |  |  |  |  |
| --- | --- | --- | --- | --- | --- | --- |
| Bone measurements in CHOP wild type and CHOP null mice | | | | | | |
|  | **Skull length** | **ICD** | **pelvis** | **femur** | **tibia** |  |
| **CHOP +/+** | 1.94±0.02 | 0.63±0.01 | 1.19±0.04 | 0.86±0.03 | 1.26±0.02 | **3 weeks** |
| **CHOP -/-** | 1.92±0.03 | 0.58±0.01 | 1.18±0.02 | 0.89±0.03 | 1.27±0.01 |  |
| **CHOP +/+** | 2.08±0.02 | 0.71±0.01 | 1.60±0.02 | 1.08±0.03 | 1.49±0.02 | **6 weeks** |
| **CHOP -/-** | 2.09±0.03 | 0.68±0.01 | 1.58±0.03 | 1.09±0.04 | 1.54±0.03 |  |
| **CHOP +/+** | 2.30±0.02 | 0.74±0.01 | 1.68±0.02 | 1.19±0.02 | 1.58±0.02 | **9 weeks** |
| **CHOP -/-** | 2.19±0.04 * | 0.77±0.01 | 1.71±0.02 | 1.19±0.03 | 1.64±0.02 |  |

|  |  |  |  |  |  |
| --- | --- | --- | --- | --- | --- |
