# Supplementary material for: Abnormal Chondrocyte Apoptosis in the Cartilage Growth Plate is Influenced by Genetic Background and Deletion of CHOP in a Targeted Mouse Model of Pseudoachondroplasia
Source: PLoS One. 2014 Feb 18;9(2):e85145. doi: 10.1371/journal.pone.0085145 (PMC3928032; doi:10.1371/journal.pone.0085145)
Supplement: Table S2 — Raw data for bone measurements in COMP m/m CHOP +/+ and COMP m/m CHOP −/− mice (n = 10, One Way ANOVA). Standard error of the mean. Key: ICD inner canthal distance, +/+ wild type, −/− knockout (null), m/m homozygous mutant. * P<0.05, ** P<0.01. (DOCX) [file pone.0085145.s007.docx]

| **Table S2** | |  |  |  |  |  |
| --- | --- | --- | --- | --- | --- | --- |
| Bone measurements in COMP m/m CHOP +/+ and COMP m/m CHOP -/- mice | | | | | | |
|  | | | | | | |
|  | **Skull length** | **ICD** | **pelvis** | **femur** | **tibia** |  |
| **COMP m/m CHOP +/+** | 1.95±0.06 | 0.62±0.02 | 1.13±0.03 | 0.84±0.03 | 1.26±0.03 | **3 weeks** |
| **COMP m/m CHOP -/-** | 1.88±0.04 | 0.61±0.01 | 1.09±0.02 | 0.85±0.02 | 1.25±0.02 |  |
| **COMP m/m CHOP +/+** | 2.22±0.02 | 0.69±0.01 | 167±0.03 | 1.11±0.04 | 1.58±0.03 | **6 weeks** |
| **COMP m/m CHOP -/-** | 2.06±0.08 | 0.68±0.01 | 1.66±0.02 | 1.04±0.02 * | 1.56±0.01 |  |
| **COMP m/m CHOP +/+** | 2.35±0.08 | 0.73±0.01 | 1.72±0.05 | 1.32±0.05 | 1.67±0.03 | **9 weeks** |
| **COMP m/m CHOP -/-** | 2.16±0.04 ** | 0.73±0.01 | 1.74±0.02 | 1.12±0.07 ** | 1.61±0.03 ** |  |
