# Supplementary material for: Abnormal Chondrocyte Apoptosis in the Cartilage Growth Plate is Influenced by Genetic Background and Deletion of CHOP in a Targeted Mouse Model of Pseudoachondroplasia
Source: PLoS One. 2014 Feb 18;9(2):e85145. doi: 10.1371/journal.pone.0085145 (PMC3928032; doi:10.1371/journal.pone.0085145)
Supplement: Table S3 — Densitometry measurement of COMP m/m CHOP +/+ and CHOP −/− Western blots at 3 weeks (n = 4, t-test). Standard error of the mean. Key: +/+ wild type, −/− knockout (null), m/m homozygous mutant. * P<0.05. (DOCX) [file pone.0085145.s008.docx]

| **Table S3** | |  | |
| --- | --- | --- | --- |
| Densitometry measurement of COMP m/m CHOP +/+ and CHOP -/- Western blots at 3 weeks | | | |
|  | **BiP (relative to GAPDH)** | | **Bcl-2 (relative to GAPDH)** |
| **COMP m/m CHOP +/+** | 0.71±0.06 | | 0.70±0.06 |
| **COMP m/m CHOP -/-** | 1.19±0.13 * | | 1.07±0.12 * |
