# Supplementary material for: Abnormal Chondrocyte Apoptosis in the Cartilage Growth Plate is Influenced by Genetic Background and Deletion of CHOP in a Targeted Mouse Model of Pseudoachondroplasia
Source: PLoS One. 2014 Feb 18;9(2):e85145. doi: 10.1371/journal.pone.0085145 (PMC3928032; doi:10.1371/journal.pone.0085145)
Supplement: Table S4 — Raw data for BrdU analysis and TUNEL analysis included in the paper (n = 3, One Way ANOVA). Standard error of the mean. Key: RZ resting zone, PZ proliferative zone, HZ hypertrophic zone, +/+ wild type, −/− knockout (null), m/m homozygous mutant. * P<0.05, ** P<0.01, *** P<0.001. (DOCX) [file pone.0085145.s009.docx]

| **Table S4** | |  |  |  |
| --- | --- | --- | --- | --- |
| Raw data for BrdU and TUNEL analysis included in the paper | | | |  |
|  | **% BrdU positive cells** | **% TUNEL positive cells in RZ** | **% TUNEL positive cells in PZ** | **% TUNEL positive cells in HZ** |
| **COMP +/+** | 8.04±0.17 | 0.26±0.09 | 0.01±0.00 | 0.89±0.36 |
| **COMP m/m** | 6.11±0.29 ** | 0.65±0.12 * | 0.10±0.03 * | 2.98±0.47 ** |
| **COMP +/+ C57+** | 9.43±0.13 | 0.76±0.27 | 0.03±0.01 | 1.43±0.50 |
| **COMP m/m C57+** | 6.66±0.10 *** | 2.02±0.67 * | 0.44±0.01 *** | 2.68±0.36 * |
| **COMP m/m CHOP +/+** | 10.26±0.47 | 3.93±0.63 | 0.27±0.05 | 9.21±0.72 |
| **COMP m/m CHOP -/-** | 9.62±0.38 | 1.36±0.26 *** | 0.24±0.04 | 8.74±0.90 |
